# A Flexible Ammonia Gas Sensor Based on a Grafted Polyaniline Grown on a Polyethylene Terephthalate Film

**DOI:** 10.3390/s24113695

**Published:** 2024-06-06

**Authors:** Masanobu Matsuguchi, Kaito Horio, Atsuya Uchida, Rui Kakunaka, Shunsuke Shiba

**Affiliations:** 1 Department of Applied Chemistry, Graduate School of Science and Engineering, Ehime University, Bunkyo-cho 3, Matsuyama 790-8577, Japan; 2 Advanced Materials Research Laboratory, NiSiNa Materials Co., Ltd., 2-6-20-3, Kitagata, Kita-ku, Okayama 700-0803, Japan

**Keywords:** NH_3_ gas sensor, polyaniline (PANI), graft polymerization, flexible sensor, room temperature operation

## Abstract

A novel NH_3_ gas sensor is introduced, employing polyaniline (PANI) with a unique structure called a graft film. The preparation method was simple: polydopamine (PD) was coated on a flexible polyethylene terephthalate (PET) film and PANI graft chains were grown on its surface. This distinctive three-layer sensor showed a response value of 12 for 50 ppm NH_3_ in a dry atmosphere at 50 °C. This value surpasses those of previously reported sensors using structurally controlled PANI films. Additionally, it is on par with sensors that combine PANI with metal oxide semiconductors or carbon materials, the high sensitivity of which have been reported. To confirm our film’s potential as a flexible sensor, the effect of bending on the its characteristics was investigated. This revealed that although bending decreased the response value, it had no effect on the response time or recovery. This indicated that the sensor film itself was not broken by bending and had sufficient mechanical strength.

## 1. Introduction

Ammonia (NH_3_) gas, a common air pollutant, is expected to be an important energy source in the coming hydrogen-fueled society [1,2,3,4]. In such applications, NH_3_ gas needs to be monitored for leak detection due to its toxicity and its adverse effects on the environment. In addition, NH_3_ gas is known to be a medical biomarker for kidney disorders and stomach infections induced by *Helicobacter pylori* [5]. Therefore, ammonia gas at sub-ppm levels is of great importance for medical diagnosis [6,7,8,9]. For these purposes, there is a need to develop a simple, low-cost NH_3_ gas sensor that does not require heating. Since it functions at room temperature, the sensor allows battery operation even in places without electrical outlets. In addition to these characteristics, in recent years, demand has grown for flexible sensor elements, which enable the sensors to be attached to clothing or complex surfaces. Such elements will further expand the application range of gas sensors generally [9,10,11,12].

Polyaniline (PANI) has the potential to meet those requirements and has long been studied as an ammonia-gas-sensing material due to its unique doping/dedoping chemistry and good environmental stability [13,14,15]. In addition, PANI is a stable electrical conductor at room temperature, which is advantageous for reducing sensor power consumption and improving portability. PANI can be synthesized easily via the chemical oxidative polymerization of aniline hydrochloride. However, sensors made by directly casting the resulting insoluble PANI dispersion onto a sensor substrate have not demonstrated sensing characteristics sufficient for practical use. This is because the formed film possesses a granular structure in where PANI chains are aggregated, resulting in poor gas diffusivity [16]. Forming a thin film is an effective way to reduce the sensor response time by reducing the diffusion depth. Similar to other conducting polymers, PANI thin films can be prepared via electrochemical polymerization and used in gas-sensing applications. Korent et al. electrochemically deposited PANI via cyclic voltammetry on screen-printed electrodes (SPEs) prepared via Au sputtering. The fabricated sensor showed excellent repeatability, reproducibility, and sensitivity [17]. However, this electrochemical polymerization method is problematic. For example, the sensor substrate is limited to electrically conductive materials and requires various special apparatuses, such as electrodes, electrochemical cells, and potentiostats/galvanostats. In addition, it is difficult to make a film with a large surface area, which makes it unsuitable for mass production.

Conventionally, many reports have addressed the complexation of PANI with nanomaterials, such as metal oxides and carbon materials, to improve sensitivity [18,19,20,21,22,23,24]. Meanwhile, it has been widely reported that gas-sensing properties can be improved through the development of structure-controlled PANI films with large surface areas, such as nanofibers [15,25], nanotubes [26,27,28,29], and networks [30], which facilitate good gas diffusivity. Sensor properties can also be enhanced by the formation of core–shell-type spherical particles [31] and hollow spherical particles with PANI on the surface layer [32,33]. In those reports, the spherical particles were in the sub-micrometer range. In contrast, we reported that it was possible to detect 46 ppb of NH_3_ gas by forming particles in the micrometer range [34].

In this paper, we propose a sensor using PANI with a unique structure called a graft film to improve NH_3_ gas-sensing properties. As discussed in detail later, the possible advantages of the graft structure are that small gas molecules can more easily diffuse between polyaniline chains, thereby increasing the opportunities for interaction between those molecules and individual polymer chains, resulting in improved sensing characteristics. In addition, the polyaniline chains are fixed to the substrate, which is expected to give the film excellent mechanical and long-term stability. However, there are very few reports on the use of polyaniline graft film as a gas-sensing film. This is because there are limited ways to chemically bond polyaniline to the sensor substrate and because the types of sensor substrates are limited. Sutar et al. reported the modification of silicone substrate surfaces with a self-assembled amino-silane monolayer; the amino groups served as reaction initiation sites for the chemical polymerization of polyaniline [35]. We propose a new, facile method for fabricating PANI graft film gas sensors that can be adapted to any type of substrate material. We then discuss the prepared PANI graft films and their NH_3_ gas-sensing characteristics.

## 2. PANI Graft Polymers

**Figure 1:**
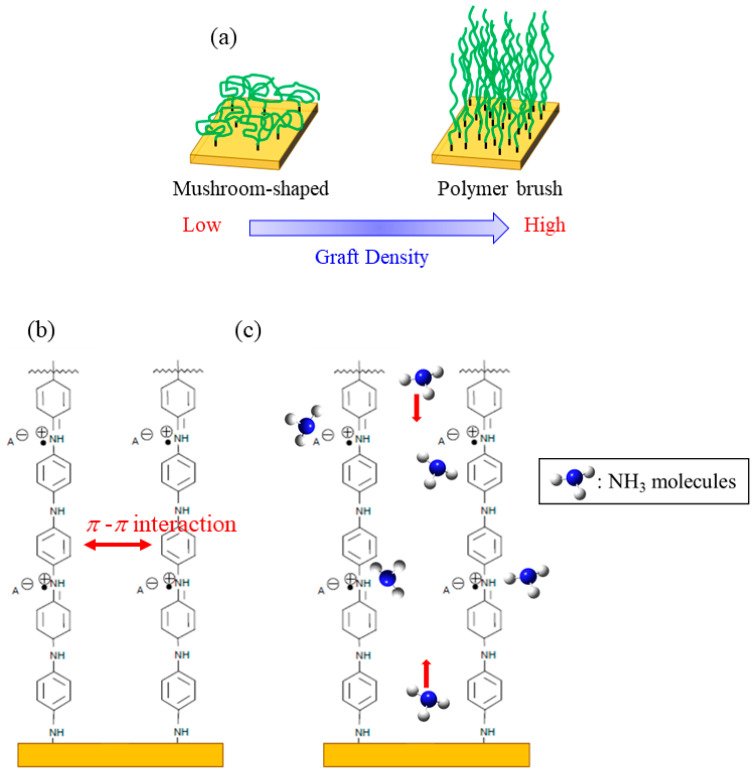
Illustrations of (**a**) graft polymer conformations, (**b**) grafted PANI chains, and (**c**) the diffusion of ammonia molecules within the PANI graft chains.

A grafted polymer is defined as an assembly of polymer chains tethered to the surface by the end of each chain. The structure usually depends on the grafting density, as shown in Figure 1a [36]. When the density is low and the interaction between the polymer chains is minimal, the conformation of the polymer chains contracts, forming a mushroom-shaped structure. On the other hand, the high density allows for interaction between the polymer chains, forcing them to stretch away from the substrate surface. This is called a “polymer brush” structure. We expect PANI to adopt this structure even at lower grafting densities due to its linear and rigid backbone structure and the strong *π*-*π* interactions between them (Figure 1b). The low-density brush structure is expected to promote gas diffusion between the polyaniline chains, as shown in Figure 1c.

**Figure 2:**
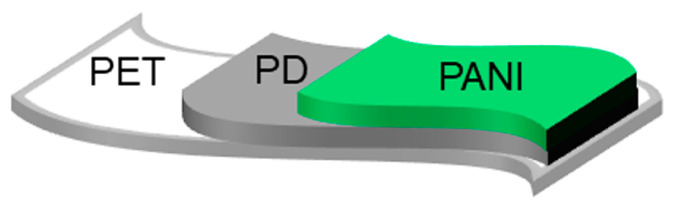
Three-layered structure of the flexible polyaniline graft film sensor.

As mentioned above, the preparation of polyaniline graft polymer requires primary amino groups on the surface as reaction-initiating groups. However, since it is impossible to react the aniline monomer with a polyethylene terephthalate (PET) film directly, we proposed a sensor with the three-layered structure shown in Figure 2. This sensor comprises the PET film, a polydopamine (PD) thin film firmly adhered to its surface, and the PANI graft film grown on the PD surface through surface-initiated polymerization. In particular, PD plays an important role in PANI graft film preparation. One of the key properties of PD is its strong adhesion to virtually any shape and size of organic or inorganic material via a simple dip-coating process [37,38]. In addition, the PD structure includes many functional groups, such as amine, imine, and catechol. These functional groups can be used to modify the surface in a variety of ways. With regard to PANI, although it was used for biomedical purposes rather than gas sensors, there is a report dealing the coating of magnetic nanoparticles with PD and the growth of high-density PANI brushes on the surface [39].

## 3. Materials and Methods

### 3.1. Materials

PET film (Teijin G2, 38 µm) was used as the sensor substrate. After ultrasonic cleaning in ethanol before use, the film was irradiated with UV light (UV Ozone Cleaner, UV-253E, Filgen, Nagoya, Japan) for 30 min to make the surface hydrophilic. Figure S1 shows the results of the contact angle measurements of the PET films before and after UV irradiation. Before irradiation, the contact angle with water was 81.6°, which was quite hydrophobic, but after irradiation, it was 34.2°, clearly indicating that the surface had become hydrophilic. Dopamine hydrochloride (95%) and *p*-phenylenediamine (97%) were purchased from Fujifilm Wako Pure Chemical Corp., Osaka, Japan and used to introduce polymerization-initiating NH_2_ groups onto the PET substrate surface. Aniline (ANI, 99%) as a monomer, ammonium persulfate (APS, 98%) as an oxidizing agent, and hydrochloric acid (HCl, 35~37.9%) and sulfuric acid (H_2_SO_4_, %) as dopants were purchased from Fujifilm Wako Pure Chemical Corp., Osaka, Japan and used to grow PANI chains.

### 3.2. Preparation of PANI Grafted on PET Film

**Figure 3:**
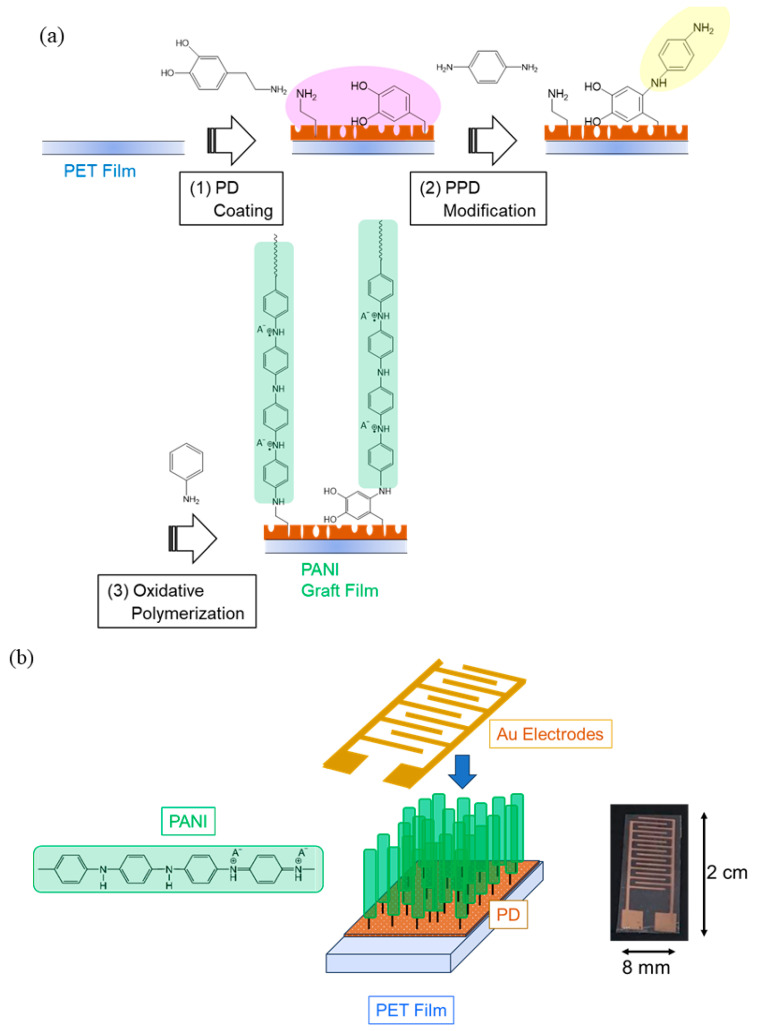
Illustration of PANI graft film sensor preparation: (**a**) polyaniline graft film; (**b**) sensor structure.

The preparation of the grafted PANI on the PET film involved in three steps, as shown in Figure 3a. First, a PD layer was deposited onto the PET film via the self-polymerization of the dopamine monomer under alkaline conditions (pH = 8.5). Under alkaline conditions, catechol was converted into a quinone, which can react with nucleophiles such as amines to form covalent bonds [37]. Thus, in the second process, the film was immersed in *p*-phenylenediamine solution at pH = 8.5. This process enabled the modification of the PD surface with *p*-phenylenediamine to increase the number of surface amino groups, serving as the initiation points for subsequent surface-initiated polymerization. Third, the oxidative graft polymerization of the aniline monomer was performed according to the literature [39]. PANI chains were grown on the PD surface by immersing the surface-modified PET substrate with amino groups in a solution containing aniline monomer and hydrochloric acid. This was followed by the addition of an aqueous APS solution and stirring at 30 °C for 24 h. Deposited PANI that was not grafted onto the PET film was dedoped once and thoroughly washed with N-methyl-2-pyrrolidone (NMP). This was because dedoped PANI dissolves in NMP, whereas chemically bonded graft PANI does not. The PANI-grafted PET film was then immersed in 1 M sulfuric acid for 2 h and redoped with sulfuric acid, followed by washing with acetone and drying under reduced pressure at room temperature. Finally, the sensor was fabricated via the vacuum deposition of interdigitated gold electrodes on the PANI graft film, as shown in Figure 3b.

The above procedures may seem very complicated, but in reality, they simply involve the repeated immersion of the substrate in the reaction solution and washing. A feature of this method is that the graft film can be produced via such a simple operation.

### 3.3. Characterization of PANI Graft Film

The surface and cross-sectional morphologies of the prepared films were characterized via field-emission scanning electron microscopy (FE-SEM, S-500, Hitachi High-Technologies, Tokyo, Japan) and atomic force microscopy (AFM, MultiMode VS-HT905SPM, Veeco Japan, Yokahama, Japan). The samples for AFM and SEM observation were prepared in the same way as the sensor. However, the sample for cross-sectional SEM observation was prepared by growing PANI graft film on a gold film sputtered on a hard silicon substrate, because cutting the flexible PET film would result in the crushed cross-section that could not be observed. The contact angle of the water droplet was measured using a contact angle meter (LSE-ME2, Nick, Kawaguchi, Japan). The water droplet volume was 1.0 µL.

### 3.4. NH_3_ Gas-Sensing Measurement

A pair of interdigitated gold electrodes was vacuum-deposited on the surface of the polyaniline graft film to prepare the sensor element, as shown in Figure 3b. Each sensor was positioned in a vessel at 30 °C or 50 °C, and dry N_2_, followed by NH_3_ gas at different concentrations diluted with dry N_2_, was introduced into it. The experimental setup is shown in Figure S2. The current was measured using an electrometer under an applied DC voltage of 1 V. An example of the sensor response to NH_3_ gas is shown in Figure S3 to illustrate the sensor characteristics. After steady resistance was obtained in a dry N_2_ atmosphere, 250 ppm of NH_3_ gas was introduced into the measurement vessel. At this time, the resistance value just before the introduction of NH_3_ gas was defined as *R*_0_. The time dependence of the resistance was then recorded while the sensor was exposed to NH_3_ gas for 30 min. The resistance increased as the sensor was exposed to NH_3_ gas, and the resistance after 30 min was defined as *R*_NH3_. This figure also shows the recovery curve of the response. After the sensor was exposed to NH_3_ gas, the atmosphere was changed back to dry N_2_, resulting in the recovery of the resistance to the original value. The response value *S* was calculated to be *S* = Δ*R*_NH3_/*R*_0_, where Δ*R*_NH3_ = *R*_NH3_ − *R*_0_. The response time *t*_80_ represented the time required to reach 80% of the total resistance change Δ*R*_NH3_, and the recovery rate *Re* was calculated as the percentage of the resistance change Δ*R*_N2_ after returning to nitrogen for 30 min of the total resistance change Δ*R*_NH3_.

## 4. Results and Discussion

### 4.1. Morphological Characterization of the PANI Graft Film

**Figure 4:**
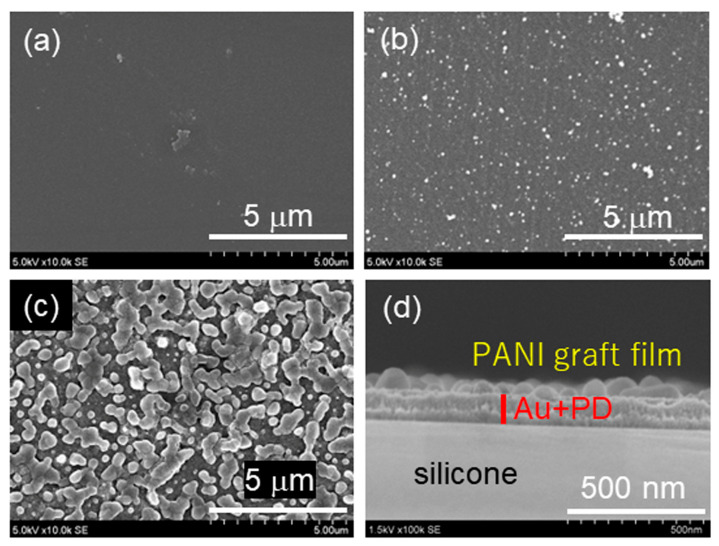
SEM images of the surfaces of (**a**) PET film, (**b**) PD-coated PET film, (**c**) PANI grafted on PET film, and (**d**) cross-section of PANI grafted film.

To confirm whether the PANI graft film had grown on the PET substrate, FT-IR measurements were first performed. However, since the coverage of the PANI graft film was relatively low, the presence of the film could not be confirmed due to the strong absorption of the PET substrate. Therefore, surface and cross-sectional SEM observations were performed to investigate the growth process and morphology of the PANI graft film, and the images are shown in Figure 4. The PET substrate surface was flat, but after PD coating, roughness became visible. In addition, after the polymerization of PANI, larger irregularities spread in a patchy manner, suggesting that polyaniline graft chains grow over the substrate starting from the unevenly coated PD. The cross-sectional SEM observation is shown in Figure 4d. It should be noted here that, as mentioned in the Experimental section, the PANI graft film for the cross-sectional observation was developed on the PD-coated gold film sputtered on the silicon substrate. The image shows that the sputtered gold layer and the PD layer on the silicon substrate have a combined thickness of about 100 nm, with a PANI layer with an about 50 nm thickness growing on them unevenly. This result is consistent with the surface SEM results and AFM images shown in Figure S4. From this result, assuming that the PANI chains extend vertically and the length of the aniline monomer is 0.4155 nm [40], the degree of polymerization of PANI is calculated to be approximately 120.

### 4.2. Ammonia Gas-Sensing Response

**Figure 5:**
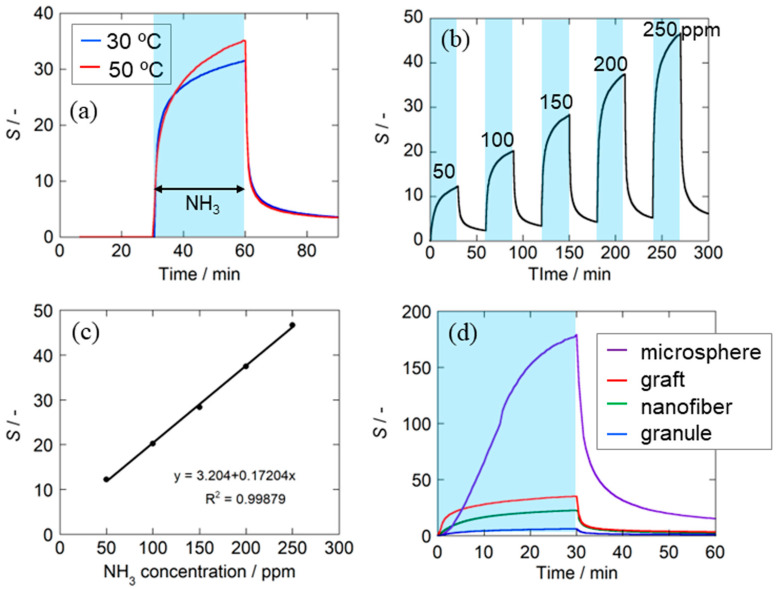
The characteristics of the PANI graft film sensor: (**a**) response–recovery curves to 250 ppm of NH_3_ measured at 30 °C and 50 °C, (**b**) response–recovery curve to 50–250 ppm NH_3_ at 50 °C, (**c**) calibration curve measured at 50 °C, and (**d**) comparison of response–recovery curves of sensors with different PANI forms.

Figure 5a displays the measurement results of the ammonia gas-sensing characteristics of the PANI graft film sensor at 30 °C and 50 °C. It was confirmed that the sensor responded reversibly at both temperatures and was operable at room temperature. This reversible response of PANI to NH_3_ is explained by a known sensing mechanism [30]. The response value *S* was 32 at 30 °C and 35 at 50 °C. In addition, the recovery rate *Re* was 89% at 30 °C and 90% at 50 °C, while the response time *t*_80_ was 7 min at 30 °C and 11 min at 50 °C, indicating that the effect of temperature on the sensor characteristics is minimal around room temperature.

A typical response of the PANI graft film sensor exposed to different concentrations of NH_3_ gas is shown in Figure 5b, and a reproducible sensor response was observed. As shown in Figure 5c, the sensor also exhibited a linear relationship between the response values and the concentration of NH_3_ in the range of 50 ppm to 250 ppm. Although our measurement equipment was unable to measure sensor responses below 50 ppm, a satisfactory response of *S* = 12 was obtained at 50 ppm, indicating that ammonia gas can be detected below 50 ppm.

The NH_3_ gas-sensing properties of sensors constructed from four different structure-controlled polyaniline films prepared in our laboratory are illustrated in Figure 5d, with the obtained sensor properties summarized and compared in Table 1. The four structure-controlled PANI films are (1) the PANI graft film in this study, (2) a PANI nanofiber film prepared using our unique on-substrate method [41], (3) a film consisting of core–shell-type spherical particles prepared via coating 4.5 µm diameter polystyrene spherical particles with a PANI shell layer [34], and (4) a film coated with a dispersion solution of granular PANIs prepared via conventional oxidative polymerization. Although the preparation conditions and measurement conditions of each sensor differ slightly, the use of structure-controlled PANI films has been shown to improve the sensor response compared with the conventional granular PANI film. The sensor based on PANI-coated polystyrene microspheres showed the highest sensitivity among the structure-controlled PANI film sensors for reasons already discussed [34]. The sensor using the present PANI graft film showed the second-largest gas response. This might be because, as expected, the grafted chain structure promotes gas diffusion between the polyaniline chains, as illustrated in Figure 1c. However, the response time and recovery rate need to further improve for practical use. In the future, it will be necessary to control the graft density and graft chain length (degree of polymerization), as well as select the optimal dopant species. Table 2 compares the response of our PANI-grafted film sensor with that of previously reported high-sensitivity polyaniline ammonia gas sensors operating at room temperature. It should be noted that some of the response values *S* in Table 2 have been recalculated to conform to our definition *S* = Δ*R*_NH3_/*R*_0_. Because the measured temperature, concentration, and humidity differ from report to report, it is difficult to make a simple comparison. In particular, it can be seen that many studies measure response characteristics under humidified conditions. It has been reported that response values are higher under humid conditions than under dry conditions [28,33,42]. Considering that our measurements were performed under dry conditions, we observed that among the reports involving sensors with structures controlled solely by PANI, our PANI graft film sensor exhibits very high response values. Furthermore, even when compared to composites of PANI with other materials, a comparable response value was obtained.

Selectivity to other gases is an important property of gas sensors, but it was not investigated in this study. However, many reports have found that sensors using PANI exhibit excellent gas selectivity to NH_3_ gas [11,18,26,30,33]. The sensor fabricated in the present research also differs only in the form of PANI, so we believe that there is essentially no problem with selectivity.

### 4.3. Mechanical Stability of PANI Graft Film Sensor

**Figure 6:**
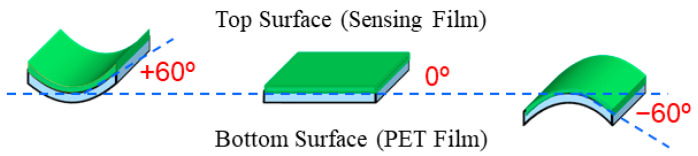
Illustration showing how the sensor is bent into shape.

**Figure 7:**
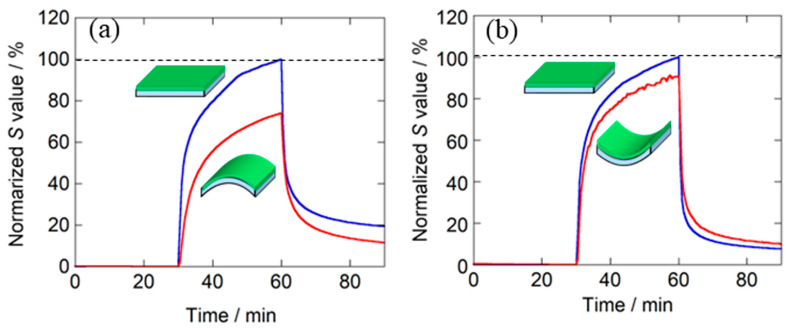
Effects of the bending angle on the response recovery curves of the PANI grafted film sensor to 250 ppm NH_3_ at 50 °C: (**a**) sensor bent 60° outward; (**b**) sensor bent 60° inward.

When considering the practical use of PANI graft film as a flexible sensor, it is necessary to investigate the effect of bending on the sensor characteristics [18,21]. In this study, as shown schematically in Figure 6, the bending angle of the sensor in the flat state was 0°. When bending 60° with the sensing surface (PANI graft film) inward, it was denoted as +60°, whereas the bending angle of 60° with the sensing surface outward was defined as −60°. The effects of these two bending angles on the sensor characteristics were measured. Figure 7 illustrates the magnitudes of the sensor response *S* when the sensor was bent in the −60° and +60° directions. Note that the vertical axis of the graph is a value normalized by setting the *S* value before the same sensor to be bent, i.e., at 0°, to 100%. The results showed that when the substrate was bent outward (−60°), the response value decreased to about 74%. On the other hand, when the sensor was bent inward (+60°), the rate of decrease was suppressed to about 10%. Thus, outward bending has a greater effect on the response than inward bending. To consider the reason for the decrease in response when the sensor was bent, the other sensor characteristics obtained are summarized in Table 3. In this table, the sensor tested when bent outward is called Sensor 1, and the sensor tested when bent inward is called Sensor 2. The measurement results showed that the sensor response value decreased due to bending, but the response time and recovery rate were not affected. This result indicates that bending did not damage the sensor. In fact, previous reports have clearly shown that the bending of flexible PANI sensors does not cause significant damage to the PANI itself [18,21]. On the other hand, an increase in the electrical resistance *R*_0_ of the sensor was observed regardless of its bending direction. This suggests that the cause of the decrease in response is the fact that bending reduces the contact between the electrode and the sensing surface. As shown in Figure 3b, the structure of this sensor has interdigitated gold electrodes placed on top of the PANI graft film. This structure was selected for two reasons. One reason is the insulating nature of the PD-coated PET surface and the growth of PANI on the layer: there was a concern that if the electrodes were placed on the PET surface, it would be impossible to make electrical contact with the PANI. The other reason is that we were concerned that the electrodes on the PET surface would be eroded by the immersion of the PET film in the reaction solution. However, due to this electrode structure, there was inadequate adhesion between the PANI graft film and the electrode. When the sensor was bent, the contact deteriorated, likely leading to an increase in the electrical resistance value *R*_0_ and a decrease in the response value. However, it is not clear at this time why the decrease in the response value was greater when the sensor was bent inward. Thus, the optimal placement of the electrodes will need to be considered in the future.

## 5. Conclusions

We fabricated an ammonia gas sensor using PANI, featuring a unique structure called graft film, as a sensing film. The fabrication method involved initially coating the sensor substrate with PD, then introducing surface NH_2_ groups using *p*-phenylenediamine, and finally conducting oxidative polymerization of an aniline monomer using these NH_2_ groups as initiation points. This method, which we developed, offer the advantage of being easy to fabricate and allows for the preparation of PANI graft films on any substrate. In this study, we used flexible PET film as the substrate. Compared to other structurally controlled PANI films and PANI composite films, the graft film sensor exhibited a significant response value of *S* = 12 at 50 ppm at 50 °C. We believe that this is explained by the unique structure of the grafted chains creating spaces between the PANI chains, making it easier for NH_3_ molecules to diffuse. Though the response time and recovery rate still need to be improved, we believe that these problems will be solved in the future by controlling the graft density and selecting the optimal dopant species. This study is the first step in a unique attempt to develop an ammonia gas sensor using the PANI graft film as NH_3_ gas-sensing agent, with properties such as gas selectivity and long-term stability still under investigation from a practical point of view. However, in this study, we were able to demonstrate the usefulness of grafted PANI as an NH_3_ gas-sensing film for operation at room temperature.

In addition, by selecting the PET film as the sensor substrate, we investigated the possibility of using it as a flexible sensor. The results confirmed that the response value decreased when the sensor was bent. This is attributed to poor contact between the PANI graft film and the electrodes, so we would like to improve the sensor structure, including the location of the electrodes, in the future. However, since bending did not affect the response time or recovery rate, we believe that there was no destruction of the sensor membrane structure itself. The graft film fabrication technology presented here is suitable for the fabrication of other flexible sensors because polydopamine can adhere to any material surface, serving as a starting point for covalent modification with desired molecules. In addition, the polymer chains are fixed to the substrate, and excellent mechanical strength is expected even when the film is bent or stretched.

## Figures and Tables

**Table 1:** The relationship between the structure of the polyaniline film and its gas-sensing characteristics to 250 ppm NH_3_.

Structure	Dopant	C_NH3_/ppm	*T*/°C	*S*/-	*t*_80_/min	*Re*/%
Graft	H_2_SO_4_	250	50	35	11	90
Nanofiber	HCl	500	50	23	9	89
Microsphere	H_2_SO_4_	250	30	179	19	92
Granule	H_2_SO_4_	250	50	6	15	85

**Table 2:** A comparison of response values between the PANI grafted film sensor and previously reported room-temperature-sensitive polyaniline ammonia gas sensors.

Material	Structure	*C*_NH3_/ppm	*T*/°C	Humidity/%RH	*S* ^1^/-	Ref.
PANI	Graft	100	50	dry	20	This work
PANI	Film	40	20 ± 3	30	4.2	[43]
PANI	Nanorod	50	27	50	1.2	[44]
PANI	Nanotube	50	25	40	27	[27]
PANI/(PE-co-GMA)	Nanofiber	25	26 ± 1	25 ± 3	25	[45]
PVDF@PANI	Core–shell	10	25	50	2	[31]
In_2_O_3_@PANI	Core–shell	100	20	50	45	[23]
GO/PANI	Hollow Rambutan	100	20	25	31	[33]
WO_3_@PANI	Nanoplate	100	25	40	33	[22]
PANI/TiO_2_	Tube	100	R.T.	dry	16	[46]
PANI/cellulose	Nanofiber	10	R.T.	45	5	[30]
PANI:PSS/Ti_3_C_2_T_X_	Nanoplate	10	24	20	3.9	[18]
PANI/HNTs	Nanotube	50	25	50	2.6	[26]

^1^ Some values of *S* were recalculated to match our definition S = Δ*R*_NH3_/*R*_0_.

**Table 3:** Effects of the bending angle on the sensing characteristics of the PANI grafted film sensor to 250 ppm NH_3_ at 50 °C.

	Bending Angle	log (*R*_0_/Ω)	Normalized *S* Value/%	*t*_80_/min	*Re*/%
Sensor 1	0	4.7	100	11	81
	−60	5.4	74	13	84
Sensor 2	0	4.8	100	9	92
	+60	5.4	91	9	90

## Data Availability

The original contributions presented in the study are included in the article/Supplementary Materials, further inquiries can be directed to the corresponding author.

## Supplementary Materials

The following are available online at https://www.mdpi.com/article/10.3390/s24113695/s1. Figure S1: Contact angle measurement of the PET film (a) before UV irradiation and (b) after UV irradiation; Figure S2: Illustration of the experimental setup; Figure S3: An example of sensor response to NH_3_ gas; Figure S4: AFM images of the PANI grafted on the PET film.
