# Supplementary material for: A Flexible Ammonia Gas Sensor Based on a Grafted Polyaniline Grown on a Polyethylene Terephthalate Film
Source: Sensors (Basel). 2024 Jun 6;24(11):3695. doi: 10.3390/s24113695 (PMC11175204; doi:10.3390/s24113695)
Supplement: Supplementary file 1 [file sensors-24-03695-s001.zip › sensors-3045969-supplementary.pdf]

Supplementary Information

A flexible ammonia gas sensor based on a grafted polyaniline grown on a polyethylene terephthalate film

Masanobu Matsuguchi [1,*], Kaito Horio [1], Atsuya Uchida [1], Rui Kakunaka [1] and Shunsuke Shiba [2]

[1] Department of Applied Chemistry, Graduate School of Science and Engineering, Ehime University, Bunkyo-cho 3, Matsuyama, Ehime 790-8577, Japan

[2] Advanced Materials Research Laboratory, NiSiNa materials Co. Ltd., 2-6-20-3, Kitagata, Kita-ku, Okayama, Okayama 700-0803, Japan

* Correspondence: matsuguchi.masanobu.mm@ehime-u.ac.jp; Tel.: +81-89-927-9933

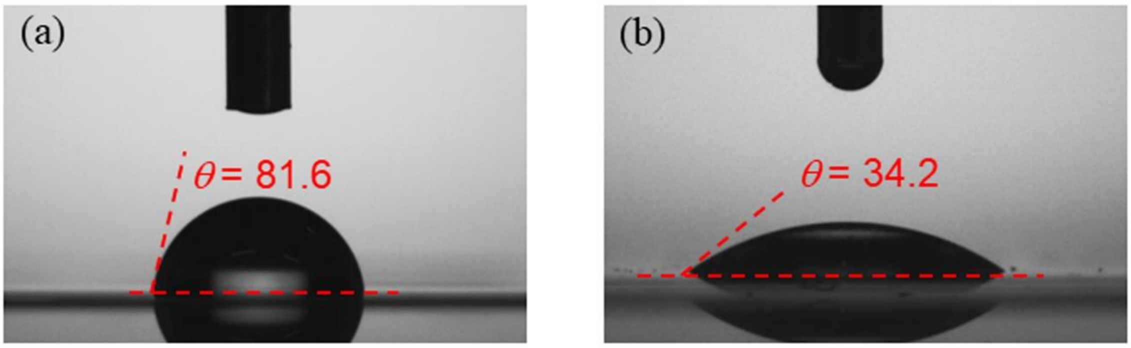

Figure S1. Contact angle measurement of PET film: (a) before UV irradiation, (b) after UV irradiation.

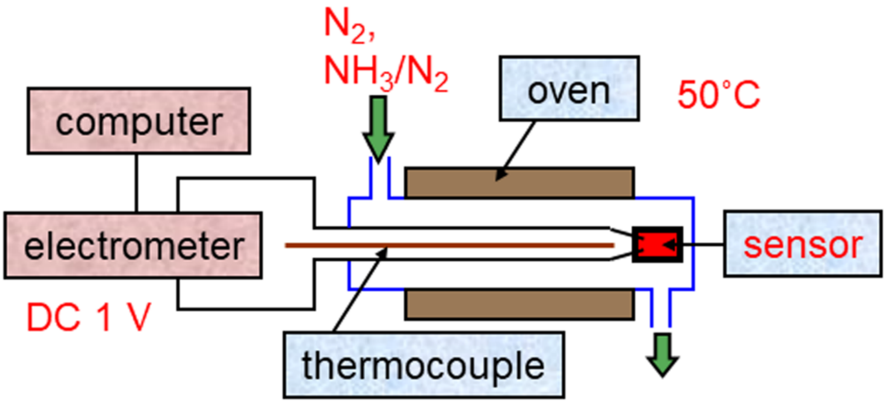

Figure S2. Illustration of experimental setup.

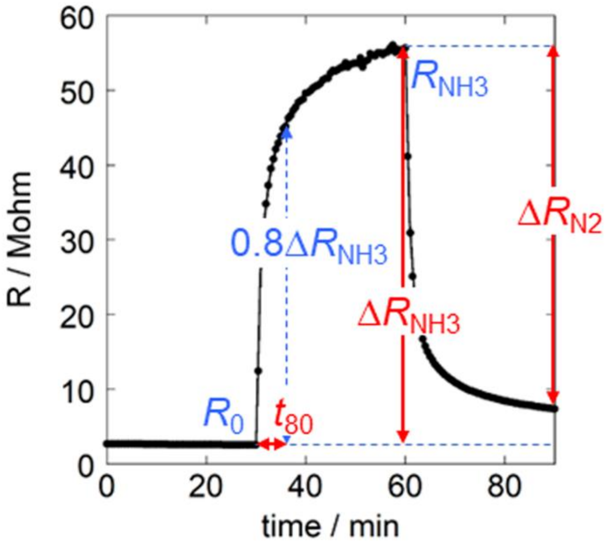

Figure S3. Example of sensor response to NH_3 gas.

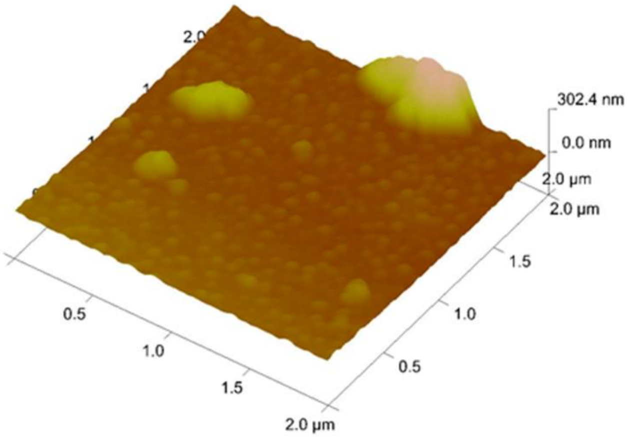

Figure S3. AFM images of the PANI grafted on PET film.
